# Product quality evaluation by confidence intervals of process yield index

**DOI:** 10.1038/s41598-022-14595-y

**Published:** 2022-06-22

**Authors:** Kuen-Suan Chen, Chang-Hsien Hsu, Kuo-Ching Chiou

**Affiliations:** 1 Department of Industrial Engineering and Management, National Chin-Yi University of Technology, Taichung, 411030 Taiwan, ROC; 2 Department of Business Administration, Chaoyang University of Technology, Taichung, 41349 Taiwan, ROC; 3 Institute of Innovation and Circular Economy, Asia University, Taichung, 41354 Taiwan, ROC; 4 Department of Business Administration, Asia University, Taichung, 41354 Taiwan, ROC; 5 Department of Finance, Chaoyang University of Technology, Taichung, 41349 Taiwan, ROC

**Keywords:** Engineering, Mathematics and computing

## Abstract

Statistical techniques have a beneficial effect on measuring process variability, analyzing the variability concerning product requirements, and eliminating the variability in product manufacturing. Process capability indices (PCIs) are not only easy to understand but also able to be directly employed by the manufacturing industry. The process yield index offers accurate measurement of the process yield, and it is a function of two unilateral six sigma quality indices. This paper initiates to develop the confidence intervals of the process yield index by using joint confidence regions of two unilateral six sigma quality indices for all quality characteristics of a product. Then integrate these joint confidence regions to find the confidence intervals of the product yield index. All manufacturing industries can use these confidence intervals to make statistical inferences to assess whether the process capability of the product and all quality characteristics has reached the required level, and to grasp the opportunities for improvement. An illustrated example on driver integrated circuit of micro hard disk is provided.

## Introduction

Process capability indices are commonly employed to assess whether the product quality can meet specifications defined in the manufacturing industry^1–3^. At the same time, the process capability indices are also commonly used in the industry, and many studies have invested in related discussions^4–6^. Based on some studies, a product usually contains multi-quality characteristics, including smaller-the-better (STB), larger-the-better (LTB), and nominal-the-best (NTB) at the same time^7,8^. Each quality characteristic needs to meet the required quality level, so that the quality of the final product can be guaranteed^9^. Moreover, numerous statisticians and quality engineers have studied process capability indices, aiming to come up with more effective methods to evaluate process potential and performance^10–12^. The six-sigma method is also a commonly used in the industry. Some studies are discussing the relationship between the six-sigma method quality level and the process capability index^13^, and then study and propose some six-sigma quality indices^14^. Two well-known unilateral six sigma quality indices, $Q_{pu}$ and $Q_{pl}$ proposed by Chang et al.^14^, are used to measure the *STB* and *LTB* quality characteristics as follows:

1 $$ Q_{pu} = \frac{USL - \mu }{\sigma }, $$

and

2 $$ Q_{pl} = \frac{\mu - LSL}{\sigma }, $$

where $USL$ is the upper specification limit, $LSL$ is the lower specification limit, $\mu$ is the process mean, and $\sigma$ refers to the process standard deviation. In normal condition, the process yield ($\% Yield$) and unilateral six sigma quality indices $Q_{pu}$ and $Q_{pl}$ have close relations displayed below:

3 $$ STB:\,\,\% Yield = p\left\{ {X \le USL} \right\} = p\left\{ {Z \le \frac{USL - \mu }{\sigma }} \right\} = \Phi \left( {Q_{pu} } \right) $$

and

4 $$ LTB:\,\,\% Yield = p\left\{ {X \ge LSL} \right\} = p\left\{ {Z \le \frac{\mu - LSL}{\sigma }} \right\} = \Phi \left( {Q_{pl} } \right), $$

where $\Phi$ is a standard function of the normal cumulative distribution. The process yield and the unilateral six sigma quality indices own a one-to-one relationship in mathematics. Therefore, some studies use these two unilateral six sigma quality indices to develop fuzzy quality evaluation model^15,16^ and fuzzy supplier selection model^17^.

For bilateral process capability index, Kane^5^ proposed a yield built on capability index $C_{pk}$ below:

5 $$ \begin{aligned} C_{pk} &= \frac{1}{3}Min\left\{ {Q_{pu} ,Q_{pl} } \right\} \hfill \\ &= \frac{1}{3}Min\left\{ {\frac{USL - \mu }{\sigma },\frac{\mu - LSL}{\sigma }} \right\} \hfill \\ \end{aligned}.$$

Given that $C_{pk}$ is a function of $\mu$ and $\sigma$, it simultaneously depends on $\mu$ and $\sigma$. Hence, a confidence interval for $C_{pk}$ can be obtained using a joint confidence region for these two parameters. The lower confidence bound is the minimum value of $C_{pk}$ over the region. The approximate confidence bounds of $C_{pk}$ can then be obtained^18^. Hence, the process evaluation of $C_{pk}$ cannot provide exact process capability measurement and process yield.

Therefore, Boyles^19^ has put forward a new bilateral process capability index which develops a one-to-one relation with the process yield as follows:

6 $$ \begin{aligned} S_{pk} &= \frac{1}{3}\Phi^{ - 1} \left\{ {\frac{1}{2}\Phi \left( {\frac{USL - \mu }{\sigma }} \right) + \frac{1}{2}\Phi \left( {\frac{\mu - LSL}{\sigma }} \right)} \right\} \hfill \\ &= \frac{1}{3}\Phi^{ - 1} \left\{ {\frac{1}{2}\Phi (Q_{pu} ) + \frac{1}{2}\Phi (Q_{pl} )} \right\} \hfill \\ \end{aligned}.$$

Then, process yield index $S_{pk}$ offers accurate measurement of the process yield. When $S_{pk} = c$, then the process yield is $Yield = 2\Phi \left( {3c} \right) - 1$. For processes of the normal distribution, the number of non-conformities is 2700 ppm, corresponding to a capable process with $S_{pk} = 1.0$.

Index $S_{pk}$ has been widely used to judge whether the process quality can meet specifications and exactly measure the process yield. For example, Lee et al.^20^ have proposed an asymptotic distribution for an estimator $\hat{S}_{pk}$. The asymptotic distribution of $\hat{S}_{pk}$ is functional in statistical inferences for $S_{pk}$. Huang et al.^8^ have applied process yield index $S_{pk}$ to assess the product quality of a backlight module with multiple process characteristics. Whereas, first, the integrated product capability ($S_{pk}^{T}$) of the backlight module is defined and, second, the individual process quality for each quality characteristic is determined. In addition, Chen et al.^9^ have considered generalizing the process yield index $S_{pk}$ targeted at processes with multiple quality characteristics. Wang and Du^21^ used index $S_{pk}$ to assess the performance of the supply chain. According to index $S_{pk}$, Wang et al.^22^ developed a new index to assess the measurement of the yield for a multiple-stream process. Lin and Pearn^23^ identified the problem of process selection with $S_{pk}$ so as to compare two processes as well as choose the one with a better production yield.

Unfortunately, the sampling distribution of yield index $S_{pk}$ is pretty complicate, and it is not easy to derive the confidence interval of the process yield index $S_{pk}$. To overcome this difficulty, we have adopted several existing techniques to construct the confidence bounds for $S_{pk}$^24–31^. For example, Chen^24^ used the bootstrap simulation technique to figure out four approximate lower confidence limits of the yield index $S_{pk}$. Shu and Wu^28^ developed a useful method to gain the fuzzy estimate of the process yield index $S_{pk}$ for measuring the manufacturing process yield. Wu et al.^30^ proposed a generalized confidence intervals for $S_{pk}$ to assess the process yield. However, most of the confidence bound studies for $S_{pk}$ are complicated and approximate estimation.

The yield index $S_{pk}$ is a function of indices $Q_{pu}$ and $Q_{pl}$. Based on the Kushler and Hurley^18^ method constructing the minimum of $C_{pk}$ over the region of the lower confidence bound, this paper initiates to develop the confidence regions of yield index $S_{pk}$ by using the joint confidence regions of the two indices $Q_{pu}$ and $Q_{pl}$. This research aims to appraise the individual process quality of a multiple-process product according to the confidence intervals of the process yield index $S_{pk}$. The method suggested by this study is a simple and intuitive tool. Then quality engineer can evaluate the process capability of the product and all quality characteristics, and decide whether to carry out process improvement. Therefore, the quality evaluation model in this paper can help the industry to improve.

## Confidence intervals

Many studies have suggested that companies use control charts to perform process control. If the process is under statistical process control, then the process capability will be evaluated^32,33^. It is assumed that each subsample contains *n* observations on quality characteristics, and there are *m* subsamples available.

In each subsample, we let $\overline{X}_{i}$ be the sample mean and $S_{i}$ be the sample variance of the *i*-*th* subsample, as displayed below:

7 $$ \overline{X}_{i} = \frac{1}{n}\sum\nolimits_{j = 1}^{n} {X_{ij} } $$

and

8 $$ S_{i} = \sqrt {\frac{1}{n - 1}\sum\nolimits_{j = 1}^{n} {\left( {X_{ij} - \overline{X}_{i} } \right)^{2} } } . $$

We define the overall sample mean and the pooled sample variance as follows:

9 $$ \overline{\overline{X}} = \frac{1}{m}\sum\limits_{i = 1}^{m} {\overline{X}_{i} } $$

and

10 $$ \overline{S} = \frac{1}{m}\sum\limits_{i = 1}^{m} {S_{i} } . $$

The estimator $\hat{Q}_{pi}$ of index $Q_{pi}$ is displayed as follows:

11 $$ \hat{Q}_{pi} = \frac{{USL - \overline{\overline{X}}}}{{\overline{s}}}\,\,\,{\text{or}}\,\,\,\,\frac{{\overline{\overline{X}} - LSL}}{{\overline{s}}} $$

For unilateral six sigma quality index $Q_{pi}$, the $100 \times \left( {1 - \alpha } \right)\%$ lower and upper confidence limits $LQ_{pi}$ and $UQ_{pi}$ for $Q_{pi}$ satisfy

12 $$ P(LQ_{pi} \le Q_{pi} \le UQ_{pi} ) = 1 - \alpha ,\,\,\,{\text{where}}\,\,i = u\,\,\,{\text{or}}\,\,\,l. $$

Based on Choi and Owen^34^, $\sqrt {mn} \times \hat{Q}_{pi}$ follows a non-central *t* distribution with $m\left( {n - 1} \right)$ degrees of freedom, where *n* is the subsample size and *m* is the number of sample groups. The non-central parameter of $\delta = \sqrt {mn} \times Q_{pi}$ is expressed as $T^{\prime}_{n - 1} \left( {\delta = \sqrt {mn} \times Q_{pi} } \right)$. Then, the above two equations can be rewritten as

13 $$ P\left[ {T^{\prime}_{n - 1} \left( {\delta = \sqrt {mn} \times Q_{Li} } \right) \le \sqrt {mn} \times \hat{Q}_{pi} } \right] = 1 - \frac{{\alpha^{\prime}}}{2} $$

and

14 $$ P\left[ {T^{\prime}_{n - 1} \left( {\delta = \sqrt {mn} \times Q_{Ui} } \right) \le \sqrt {mn} \times \hat{Q}_{pi} } \right] = \frac{{\alpha^{\prime}}}{2}, $$

where *i* $= u$ or $l$ and $\alpha^{\prime} = {\alpha \mathord{\left/ {\vphantom {\alpha q}} \right. \kern-\nulldelimiterspace} q}$; $q$ is the total number of quality characteristics for a multi-process product, $m$ is the number of sample groups, and $n$ is the sample size for each sub-sample.

Online Appendix S1 displays the lower limit ($LQ_{pi}$) and the upper limits ($UQ_{pi}$) of the 95% confidence intervals for different $Q_{pi}$ values with $n = 11$, $m = 30$, and $q = 6\left( 1 \right)10$. For instance, given sub-sample size $n = 11$, $m = 30$, $q = 6$, $\alpha = 0.05$, when $\hat{Q}_{pi} = 3.60$, from SAS program results, the $LQ_{pi}$ and $UQ_{pi}$ values are 3.222 and 3.981. The confidence intervals for different $Q_{pi}$ values can be obtained in Online Appendix S1. Online Appendix S1 only provides the numerical values which are used in this study. Different sample size, different number of sample groups or different number of quality characteristics could possibly occur in the practical application. For easier explanation, we only perform the selected sample size and selected number of sample groups and the results are listed in Online Appendix S1. The numbers of quality characteristics are computed from 6 to 10 in Online Appendix S1.

Assume there are $q$ quality characteristics in a product, the process capability index for the *j*th characteristic will become

15 $$ S_{pkj} = \frac{1}{3}\Phi^{ - 1} \left\{ {\frac{1}{2}\Phi (Q_{puj} ) + \frac{1}{2}\Phi (Q_{plj} )} \right\} $$

Let the confidence intervals of indices $Q_{puj}$ and $Q_{plj}$ are denoted by $\left( {LQ_{puj} ,UQ_{puj} } \right)$ and $\left( {LQ_{plj} ,UQ_{plj} } \right)$, respectively. And index $S_{pkj}$ is a function of indices $Q_{puj}$ and $Q_{plj}$, the confidence intervals for index $S_{pkj}$ can be described as follows.

Lower confidence interval for the $j$th characteristic ($LS_{pkj}$):

16 $$ LS_{pkj} = \frac{1}{3}\Phi^{ - 1} \left\{ {\frac{1}{2}\Phi (LQ_{puj} ) + \frac{1}{2}\Phi (LQ_{plj} )} \right\} $$

Upper confidence interval for the $j$th characteristic ($US_{pkj}$):

17 $$ US_{pkj} = \frac{1}{3}\Phi^{ - 1} \left\{ {\frac{1}{2}\Phi (UQ_{puj} ) + \frac{1}{2}\Phi (UQ_{plj} )} \right\} $$

Thus, the integrated process capability index for the entire product is

18 $$ S_{pk}^{T} = \frac{1}{3}\Phi^{ - 1} \left\{ {\frac{1}{2}\left( {\prod\limits_{j = 1}^{q} {\left[ {2\Phi (3S_{pkj} ) - 1} \right]} + 1} \right)} \right\} $$

Let the confidence intervals of indices $S_{pk}^{T}$ are denoted by $\left( {LS_{pk}^{T} ,US_{pk}^{T} } \right)$, and the confidence intervals for index $S_{pk}^{T}$ can be described as follows.

Lower confidence interval for the integrated product ($LS_{pk}^{T}$):

19 $$ LS_{pk}^{T} = \frac{1}{3}\Phi^{ - 1} \left\{ {\frac{1}{2}\left( {\prod\limits_{j = 1}^{q} {\left[ {2\Phi (3LS_{pkj} ) - 1} \right]} + 1} \right)} \right\} $$

Upper confidence interval for the integrated product ($US_{pk}^{T}$):

20 $$ US_{pk}^{T} = \frac{1}{3}\Phi^{ - 1} \left\{ {\frac{1}{2}\left( {\prod\limits_{j = 1}^{q} {\left[ {2\Phi (3US_{pkj} ) - 1} \right]} + 1} \right)} \right\} $$

As previously mentioned and based on Eqs. (16, 17, 19, 20), these confidence intervals allow statistical inferences to be made to assess whether the product's process capability and all quality characteristics are at the required level.

## Application procedures of confidence intervals

Products are commonly designed with many quality characteristics. Pearn et al.^25^ has proposed how to determine the incorporated process of a product which contains multiple processes using index $S_{pk}$. What we emphasize here is the construction and the application through the confidence intervals of the process yield index. The previous section illustrates how to calculate the confidence intervals for the incorporated process capability of a product which contains multiple processes on the basis of the process yield index $S_{pk}$.

**Figure 1:**
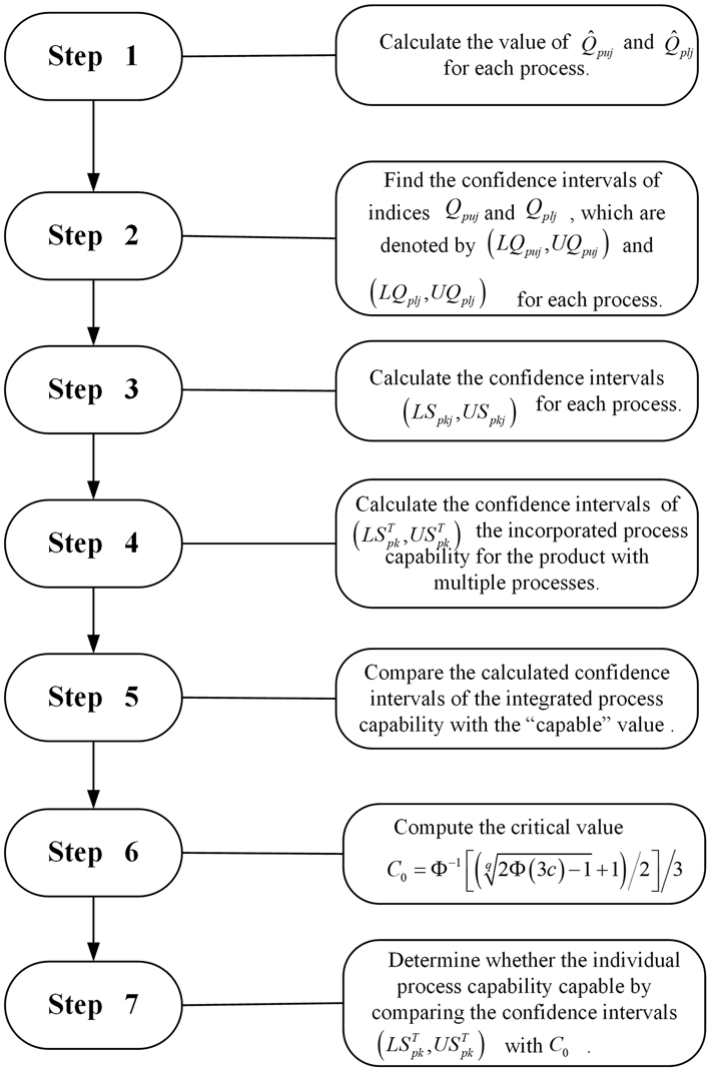
Evaluation procedure steps chart.

We assume that *q* quality characteristics in a product, *m* sample groups, and sub-sample sizes *n* are collected for each process. Since process yield index $S_{pkj}$ is a function of unilateral indices $Q_{puj}$ and $Q_{plj}$, the estimates of $Q_{puj}$ and $Q_{plj}$ for each process should be calculated. Then the confidence intervals $\left( {LQ_{puj} ,UQ_{puj} } \right)$ and $\left( {LQ_{plj} ,UQ_{plj} } \right)$ for the estimates of $Q_{puj}$ and $Q_{plj}$ are determined according to the formulas in “Application procedures of confidence intervals”. Or confidence intervals can also be found in Online Appendix S1. According to the confidence intervals for the estimates of $Q_{puj}$ and $Q_{plj}$, the confidence intervals $\left( {LS_{pkj} ,US_{pkj} } \right)$ for each process is calculated. Finally, the confidence intervals for the incorporated process capability of the product with multiple processes are determined according to the formula of $LS_{pk}^{T}$ and $US_{pk}^{T}$. For practical and easier application, the Fig. 1 evaluation procedure steps chart is shown as below.

The above evaluation steps are provided for manufacturers to follow and calculate the index confidence interval of the product and all quality characteristics, thereby evaluating whether the process capability of the product and all quality characteristics meets the quality level requirements. Next, this paper will use an example to illustrate the application of the above evaluation steps.

## An illustrative example

As the market demand on DVD player, MP3 and PDA rapidly grows, the demand on gigabyte capacity of multimedia data storage also increases. Nowadays, micro Hard Disk Driver (micro HDD) and Flash Memory card are the major products in terms of multimedia data storage. The storage capacity of a Flash Memory card ranges from 16 to 128 GB, while the storage capacity of micro HDD ranges from 500 GB to 5 TB. Driver IC is the key electronic component of micro HDD, functioning as anti-mechanical shock and read head control. In addition, it is responsible for the performance and effectiveness of micro HDD. The hard disk adopts the PCMCIA interface to connect to other equipment. To fit the design of thinner, smaller and low profile of micro HDD, the vertical dimension of driver IC gets thinner and thinner. There are six essential quality dimensions including top space, top loop, die, film, mold thickness and substrate thickness for a driver IC. The abovementioned evaluation method is adopted to assess the incorporated capability for a multi-process product, driver IC, for 1.8″ HDD with mold thickness of 0.25 mm. Table 1 shows brief information about this production line. We illustrate the above procedure step by step with a numerical example as follows:

**Table 1:** The quality characteristics and specifications for driver IC.

Mold thickness		0.25 mm	
Item	Layers	µm	Tolerance (µm)
A	Top Space	95	10
B	Top Loop	55	15
C	Die	70	5
D	Film	30	5
E	Mold thickness	250	50
F	Substrate thickness	110	25

Step 1 Calculate the estimates $\hat{Q}_{puj}$ and $\hat{Q}_{plj}$ of indices $Q_{puj}$ and $Q_{plj}$ for each process in Table 2. For example, the $\hat{Q}_{puA}$ and $\hat{Q}_{plA}$ for process A are 2.73 and 4.08, respectively.

**Table 2:** The analytical results of a driver IC process.

Item	$USL_{j}$	$LSL_{j}$	$\overline{\overline{X}}_{j}$	$\overline{s}_{j}$	$\hat{Q}_{puj}$	$LQ_{puj}$	$UQ_{puj}$	$\hat{C}_{plj}$	$LQ_{plj}$	$UQ_{plj}$	$LS_{pkj}$	*US*_*pkj*_
A*	105	85	97.0	8.8	2.73	2.43	3.033	4.08	3.66	4.503	0.889	1.079
B	70	40	52.7	12.4	4.17	3.741	4.602	3.06	2.73	3.39	0.981	1.191
C*	75	65	69.1	4.4	4.02	3.606	4.44	2.79	2.484	3.096	0.905	1.098
D*	35	25	29.4	4.8	3.51	3.141	3.882	2.76	2.457	3.063	0.886	1.084
E*	300	200	253	51	2.76	2.457	3.063	3.12	2.787	3.456	0.863	1.067
F	135	85	108	22	3.69	3.306	4.077	3.15	2.814	3.489	0.992	1.216
Lower confidence interval for the integrated product									$LS_{pk}^{T}$ $=$ 0.9626			
Upper confidence interval for the integrated product									$US_{pk}^{T}$ $=$ 0.9977
Step 2 Find the confidence intervals of indices $Q_{puj}$ and $Q_{plj}$, which are denoted by $\left( {LQ_{puj} ,UQ_{puj} } \right)$ and $\left( {LQ_{plj} ,UQ_{plj} } \right)$ for each process in Table 2. For example, the $\left( {LQ_{{pu{\text{A}}}} ,UQ_{{pu{\text{A}}}} } \right)$ and $\left( {LQ_{{pl{\text{A}}}} ,UQ_{{pl{\text{A}}}} } \right)$ for process A are $\left( {2.430,3.033} \right)$ and $\left( {3.660,4.503} \right)$, respectively.
Step 3 Calculate the confidence intervals $\left( {LS_{pkj} ,US_{pkj} } \right)$ for each process in Table 2. For example, the confidence intervals $\left( {LS_{{pk{\text{A}}}} ,US_{{pk{\text{A}}}} } \right)$ for process A are $\left( {2.667,3.237} \right)$.
Step 4 Calculate the confidence intervals for the integrated process capability for the 6-process driver IC. The confidence interval for the product is $\left( {2.888,2.993} \right)$.
Step 5 Compare the confidence intervals of driver IC with capable value, $c = 1.0$. Since the capable value does not range in the confidence intervals, the integrated process capability for the driver IC is not capable.
Step 6 There are six essential dimensions for this product. Thus from the computation of the formula $C_{0} = {{\Phi^{ - 1} \left[ {{{\left( {\sqrt[q]{{2\Phi \left( {3c} \right) - 1}} + 1} \right)} \mathord{\left/ {\vphantom {{\left( {\sqrt[q]{{2\Phi \left( {3c} \right) - 1}} + 1} \right)} 2}} \right. \kern-\nulldelimiterspace} 2}} \right]} \mathord{\left/ {\vphantom {{\Phi^{ - 1} \left[ {{{\left( {\sqrt[q]{{2\Phi \left( {3c} \right) - 1}} + 1} \right)} \mathord{\left/ {\vphantom {{\left( {\sqrt[q]{{2\Phi \left( {3c} \right) - 1}} + 1} \right)} 2}} \right. \kern-\nulldelimiterspace} 2}} \right]} 3}} \right. \kern-\nulldelimiterspace} 3}$, the critical value for individual process capability is 1.170 ($C_{0} = 1.170$).
Step 7 From Table 2, compare the required value $C_{0}$ with the confidence interval for each process and make a decision to determine which quality characteristic need to improvement. If $C_{0}$ ranges $LS_{pk}^{T}$ and $US_{pk}^{T}$, then it is concluded that the individual process capability can meet the preset target; otherwise, the conclusion will be reverse. The processes marked with an “$*$” indicate that the process capabilities are not capable.

According to the above evaluation steps, the quality engineer can complete the process capability evaluation of the product and all quality characteristics, and decide whether to carry out process improvement. From Table 2, items B and F are capable processes. Quality engineers need to launch quality enhancement projects on incapable items A, C, D and E for process capability improvement. Obviously, through the evaluation steps in this article, process engineers can simultaneously master and improve four quality characteristics with insufficient process capabilities. When the process capabilities of all quality characteristics meet the quality requirements, the product's process capabilities will meet the quality requirements.

## Conclusions

PCIs are widely employed by the manufacturing industry to evaluate whether the process capability can meet the specifications. Regarding the product with multiple processes, customers concern the incorporated capability of the product. Based on the yield index $S_{pkj}$ for quality characteristic *j*, this paper discussed the incorporated process capability of a product with multiple processes in terms of calculating the confidence intervals of the yield index $S_{pkj}$. Whereas the yield index $S_{pkj}$ is a function of indices $Q_{puj}$ and $Q_{plj}$, the confidence intervals for individual process of indices $Q_{puj}$ and $Q_{plj}$ are computed to attain the confidence intervals of $S_{pkj}$. Then integrate these confidence intervals to derive the confidence intervals of the entire product yield index $S_{pk}^{T}$. Evaluation procedures are presented in steps to assist practical application. The quality engineer can follow the evaluation procedures to complete the process capability evaluation of the product and all quality characteristics, and decide whether to carry out process improvement. The above research is based on the premise of the normal process. When the process distribution is non-normal, the method in this paper will have a large error, so it can be the focus of future research.

## Supplementary Information

Supplementary Information.
