# Supplementary material for: Product quality evaluation by confidence intervals of process yield index
Source: Sci Rep. 2022 Jun 22;12:10508. doi: 10.1038/s41598-022-14595-y (PMC9217850; doi:10.1038/s41598-022-14595-y)
Supplement: Supplementary file 1 — Supplementary Information. [file 41598_2022_14595_MOESM1_ESM.doc]

**Appendix: The lower ($LQ_{pi}$) and upper limits ($UQ_{pi}$) of the 95% confidence intervals for different $Q_{pi}$ values with $n=11$, $m=30$, and** $q=6\left(1\right)10$

| $\hat{Q}_{pi}$ | *q* = 6 | | *q* = 7 | | *q* = 8 | | *q* = 9 | | *q* = 10 | |
| --- | --- | --- | --- | --- | --- | --- | --- | --- | --- | --- |
|  | $LQ_{pi}$ | $UQ_{pi}$ | $LQ_{pi}$ | $UQ_{pi}$ | $LQ_{pi}$ | $UQ_{pi}$ | $LQ_{pi}$ | $UQ_{pi}$ | $LQ_{pi}$ | $UQ_{pi}$ |
| 2.73 | 2.43 | 3.033 | 2.424 | 3.039 | 2.418 | 3.045 | 2.412 | 3.051 | 2.409 | 3.054 |
| 2.76 | 2.457 | 3.063 | 2.451 | 3.072 | 2.445 | 3.078 | 2.439 | 3.084 | 2.436 | 3.087 |
| 2.79 | 2.484 | 3.096 | 2.478 | 3.105 | 2.472 | 3.111 | 2.466 | 3.117 | 2.463 | 3.12 |
| 2.82 | 2.511 | 3.129 | 2.505 | 3.138 | 2.499 | 3.144 | 2.493 | 3.15 | 2.49 | 3.153 |
| 2.85 | 2.538 | 3.162 | 2.532 | 3.168 | 2.526 | 3.177 | 2.52 | 3.183 | 2.517 | 3.186 |
| 2.88 | 2.568 | 3.195 | 2.562 | 3.201 | 2.553 | 3.207 | 2.547 | 3.213 | 2.544 | 3.219 |
| 2.91 | 2.595 | 3.228 | 2.586 | 3.234 | 2.58 | 3.24 | 2.577 | 3.249 | 2.571 | 3.252 |
| 2.94 | 2.622 | 3.261 | 2.616 | 3.267 | 2.61 | 3.273 | 2.601 | 3.279 | 2.598 | 3.285 |
| 2.97 | 2.649 | 3.294 | 2.643 | 3.3 | 2.637 | 3.306 | 2.631 | 3.312 | 2.625 | 3.318 |
| 3 | 2.676 | 3.324 | 2.67 | 3.333 | 2.664 | 3.339 | 2.658 | 3.345 | 2.652 | 3.351 |
| 3.03 | 2.703 | 3.36 | 2.697 | 3.366 | 2.691 | 3.372 | 2.685 | 3.378 | 2.679 | 3.384 |
| 3.06 | 2.73 | 3.39 | 2.724 | 3.399 | 2.718 | 3.405 | 2.712 | 3.411 | 2.706 | 3.417 |
| 3.09 | 2.757 | 3.423 | 2.751 | 3.432 | 2.745 | 3.438 | 2.739 | 3.444 | 2.733 | 3.45 |
| 3.12 | 2.787 | 3.456 | 2.778 | 3.465 | 2.772 | 3.471 | 2.766 | 3.477 | 2.76 | 3.483 |
| 3.15 | 2.814 | 3.489 | 2.805 | 3.498 | 2.799 | 3.504 | 2.793 | 3.51 | 2.787 | 3.516 |
| 3.18 | 2.841 | 3.522 | 2.832 | 3.528 | 2.826 | 3.537 | 2.82 | 3.543 | 2.814 | 3.549 |
| 3.21 | 2.868 | 3.555 | 2.859 | 3.561 | 2.853 | 3.57 | 2.847 | 3.576 | 2.841 | 3.582 |
| 3.24 | 2.895 | 3.588 | 2.886 | 3.594 | 2.88 | 3.603 | 2.874 | 3.609 | 2.868 | 3.615 |
| 3.27 | 2.922 | 3.621 | 2.913 | 3.627 | 2.907 | 3.636 | 2.901 | 3.642 | 2.898 | 3.648 |
| 3.3 | 2.949 | 3.654 | 2.943 | 3.66 | 2.934 | 3.669 | 2.928 | 3.675 | 2.925 | 3.681 |
| 3.33 | 2.976 | 3.684 | 2.97 | 3.693 | 2.964 | 3.702 | 2.955 | 3.708 | 2.952 | 3.714 |
| 3.36 | 3.003 | 3.717 | 2.997 | 3.726 | 2.991 | 3.735 | 2.985 | 3.741 | 2.979 | 3.744 |
| 3.39 | 3.033 | 3.75 | 3.024 | 3.759 | 3.018 | 3.768 | 3.009 | 3.774 | 3.006 | 3.777 |
| 3.42 | 3.06 | 3.783 | 3.051 | 3.792 | 3.045 | 3.798 | 3.039 | 3.807 | 3.033 | 3.81 |
| 3.45 | 3.087 | 3.816 | 3.078 | 3.825 | 3.072 | 3.831 | 3.063 | 3.84 | 3.06 | 3.843 |
| 3.48 | 3.114 | 3.849 | 3.105 | 3.858 | 3.099 | 3.864 | 3.093 | 3.873 | 3.087 | 3.876 |
| 3.51 | 3.141 | 3.882 | 3.132 | 3.891 | 3.126 | 3.897 | 3.117 | 3.906 | 3.114 | 3.909 |
| 3.54 | 3.168 | 3.915 | 3.159 | 3.924 | 3.153 | 3.93 | 3.147 | 3.939 | 3.141 | 3.942 |
| 3.57 | 3.195 | 3.948 | 3.186 | 3.957 | 3.18 | 3.963 | 3.174 | 3.972 | 3.168 | 3.975 |
| 3.6 | 3.222 | 3.981 | 3.267 | 3.99 | 3.207 | 3.996 | 3.201 | 4.005 | 3.195 | 4.008 |
| 3.63 | 3.249 | 4.014 | 3.24 | 4.02 | 3.234 | 4.029 | 3.228 | 4.035 | 3.222 | 4.041 |
| 3.66 | 3.276 | 4.047 | 3.27 | 4.053 | 3.261 | 4.062 | 3.255 | 4.071 | 3.249 | 4.074 |
| 3.69 | 3.306 | 4.077 | 3.297 | 4.086 | 3.288 | 4.095 | 3.282 | 4.101 | 3.276 | 4.107 |
| 3.72 | 3.333 | 4.11 | 3.324 | 4.119 | 3.315 | 4.128 | 3.309 | 4.134 | 3.303 | 4.14 |
| 3.75 | 3.36 | 4.143 | 3.351 | 4.152 | 3.342 | 4.161 | 3.336 | 4.167 | 3.33 | 4.173 |
| 3.78 | 3.387 | 4.176 | 3.378 | 4.185 | 3.369 | 4.194 | 3.363 | 4.2 | 3.357 | 4.206 |
| 3.81 | 3.414 | 4.209 | 3.405 | 4.218 | 3.399 | 4.227 | 3.39 | 4.233 | 3.384 | 4.239 |
| 3.84 | 3.441 | 4.242 | 3.432 | 4.251 | 3.426 | 4.26 | 3.417 | 4.266 | 3.411 | 4.272 |
| 3.87 | 3.468 | 4.275 | 3.459 | 4.284 | 3.453 | 4.293 | 3.444 | 4.299 | 3.438 | 4.305 |
| 3.9 | 3.495 | 4.308 | 3.486 | 4.317 | 3.48 | 4.326 | 3.471 | 4.332 | 3.465 | 4.338 |
| 3.93 | 3.522 | 4.341 | 3.513 | 4.35 | 3.507 | 4.359 | 3.498 | 4.365 | 3.492 | 4.371 |
| 3.96 | 3.549 | 4.374 | 3.54 | 4.383 | 3.534 | 4.392 | 3.525 | 4.398 | 3.519 | 4.404 |
| 3.99 | 3.576 | 4.407 | 3.567 | 4.416 | 3.561 | 4.425 | 3.552 | 4.431 | 3.546 | 4.437 |
| 4.02 | 3.606 | 4.44 | 3.594 | 4.449 | 3.588 | 4.458 | 3.579 | 4.464 | 3.573 | 4.47 |
| 4.05 | 3.633 | 4.473 | 3.621 | 4.482 | 3.615 | 4.491 | 3.606 | 4.497 | 3.6 | 4.503 |
| 4.08 | 3.66 | 4.503 | 3.648 | 4.515 | 3.642 | 4.524 | 3.633 | 4.53 | 3.627 | 4.536 |
| 4.11 | 3.687 | 4.536 | 3.675 | 4.548 | 3.669 | 4.557 | 3.663 | 4.563 | 3.654 | 4.569 |
| 4.14 | 3.714 | 4.569 | 3.705 | 4.581 | 3.696 | 4.59 | 3.69 | 4.596 | 3.681 | 4.602 |
| 4.17 | 3.741 | 4.602 | 3.732 | 4.614 | 3.723 | 4.623 | 3.717 | 4.629 | 3.708 | 4.635 |
| 4.2 | 3.768 | 4.635 | 3.759 | 4.647 | 3.75 | 4.656 | 3.741 | 4.662 | 3.735 | 4.668 |
